# (*Z*)-Amino­(2-methyl-3-oxoisoindolin-1-yl­idene)acetonitrile

**DOI:** 10.1107/S1600536809045681

**Published:** 2009-11-07

**Authors:** Dieter Schollmeyer, Dorota Ferenc, Till Opatz

**Affiliations:** a Institut für Organische Chemie, Universität Mainz, Duesbergweg 10-14, 55128 Mainz, Germany; b Department Chemie, Universität Hamburg, Martin-Luther-King-Platz 6, 20146 Hamburg, Germany

## Abstract

The asymmetric unit of the title compound, C_11_H_9_N_3_O, contains two independent and nearly identical mol­ecules (*A* and *B*). Mol­ecule *A* can be transformed to *B* using a rotation of approximately 85° around the [111] direction. Each *A* mol­ecule is connected to three *B* mol­ecules *via* N—H⋯N and N—H⋯O hydrogen bonds and *vice versa*. Centrosymmetric­ally related mol­ecules of the same residue form π–π inter­actions with centroid–centroid distances of 4.326 (1) and 3.826 (1) Å for the benzene rings of mol­ecules *A* and *B*, respectively.

## Related literature

For the preparation of the compound as well as the crystal structure of the corresponding 2-benzyl derivative, see: Opatz & Ferenc (2004 ▶). For the crystal structure of the distantly related compound *N*-(2-amino-1,2-dicyano­vinyl)acetamide, see: Al-Azmi *et al.*, (2001 ▶).
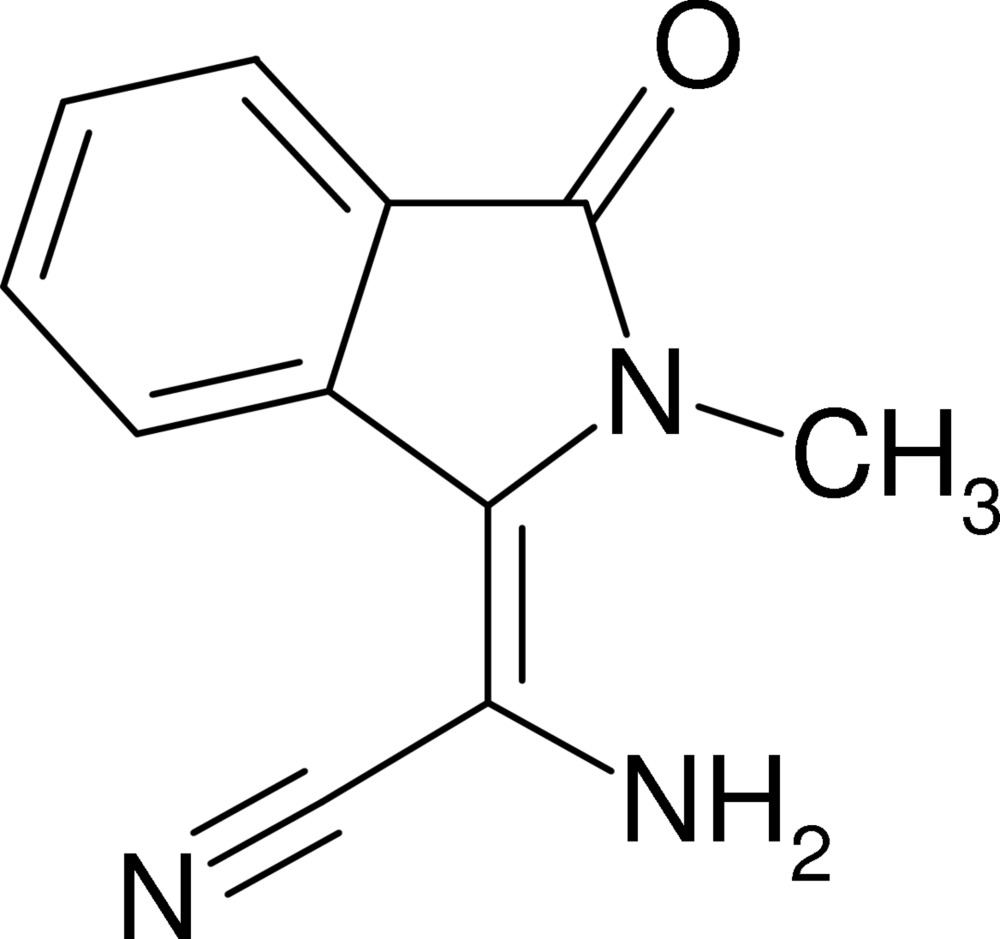


## Experimental

### 

#### Crystal data

C_11_H_9_N_3_O
*M* *_r_* = 199.21
Triclinic, 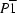

*a* = 8.5021 (7) Å
*b* = 8.5080 (6) Å
*c* = 14.0412 (11) Å
α = 80.741 (5)°
β = 80.797 (5)°
γ = 68.329 (4)°
*V* = 925.98 (12) Å^3^
*Z* = 4
Mo *K*α radiation
μ = 0.10 mm^−1^
*T* = 298 K
0.44 × 0.30 × 0.24 mm

#### Data collection

Bruker SMART APEXII diffractometer
Absorption correction: none
13710 measured reflections
4570 independent reflections
3668 reflections with *I* > 2σ(*I*)
*R*_int_ = 0.095

#### Refinement

*R*[*F* ^2^ > 2σ(*F* ^2^)] = 0.047
*wR*(*F* ^2^) = 0.114
*S* = 0.97
4570 reflections
274 parameters
H-atom parameters constrained
Δρ_max_ = 0.24 e Å^−3^
Δρ_min_ = −0.29 e Å^−3^

### 

Data collection: *APEX2* (Bruker, 2006 ▶); cell refinement: *SAINT* (Bruker, 2006 ▶); data reduction: *SAINT*; program(s) used to solve structure: *SIR97* (Altomare *et al.*, 1999 ▶); program(s) used to refine structure: *SHELXL97* (Sheldrick, 2008 ▶); molecular graphics: *PLATON* (Spek, 2009 ▶); software used to prepare material for publication: *PLATON*.

## Supplementary Material

Crystal structure: contains datablocks I, global. DOI: 10.1107/S1600536809045681/bt5125sup1.cif

Structure factors: contains datablocks I. DOI: 10.1107/S1600536809045681/bt5125Isup2.hkl

Additional supplementary materials:  crystallographic information; 3D view; checkCIF report

## Figures and Tables

**Table 1:** Hydrogen-bond geometry (Å, °)

*D*—H⋯*A*	*D*—H	H⋯*A*	*D*⋯*A*	*D*—H⋯*A*
N13*A*—H13*A*⋯N15*B* ^i^	0.97	2.08	3.035 (3)	169
N13*B*—H13*C*⋯O10*A* ^ii^	0.94	2.04	2.948 (2)	163
N13*B*—H13*D*⋯N13*A* ^iii^	0.97	2.52	3.238 (3)	131

Symmetry codes: (i) 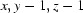; (ii) 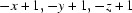; (iii) 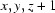.

**Fig. 1.**
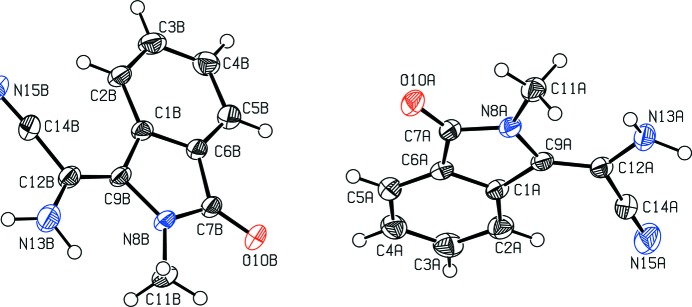
View of compound I. Displacement ellipsoids are drawn at the 50% probability level.

**Fig. 2.**
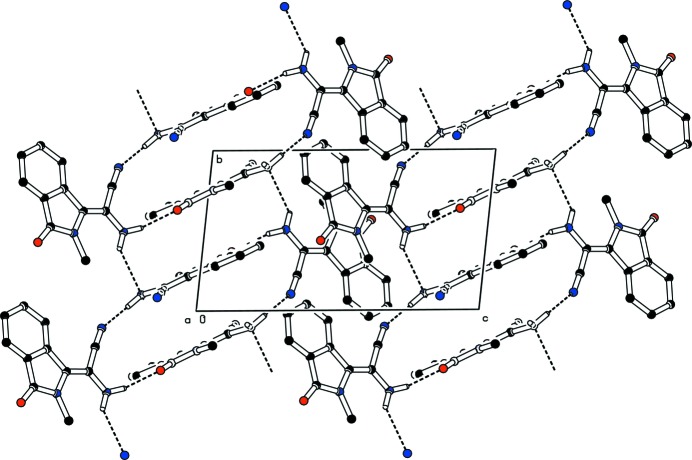
Part of the packing diagram. Hydrogen bonds with dashed lines.

## supplementary crystallographic information

### Comment

Amino(2-alkyl-3-oxo-2,3-dihydro-1*H*-isoindol-1-ylidene)acetonitriles are readily obtained in a three-component reaction between 2-carboxybenzaldehyde (2-formylbenzoic acid), aliphatic amines and cyanide in acidic medium. While steric hindrance is a decisive factor and the reaction fails for α-branched primary amines such as isopropylamine, all tested unbranched primary amines give the desired products. Consequently, the highest yield of 53% is found for the preparation of the title compound, in which unfavorable steric interactions are reduced to a minimum. Despite the fact that this compound had been obtained as the prototype example of the series, the determination of its crystal structure was hampered by twinning of the crystals. Compared to the *N*-benzyl derivative described earlier (Opatz & Ferenc, 2004), the exocyclic aminoacetonitrile unit is even less twisted against the plane of the bicyclic π-system (5.0 (1)° and 1.7 (2)°) (Fig. 1). Furthermore, the compound forms a hydrogen bonded network, in which both exocyclic nitrogen atoms as well as the oxygen atom act as acceptors and the NH_2_ group is the double donor. Centrosymetrically related molecules of the same residue form π-π-interactions. The distances between the centroids are 4.326 (1)Å and 3.826 (1)Å for the rings C1–C6 of A and B respectively (Fig. 2).

### Experimental

The preparation was carried out as described in the procedure reported by Opatz and Ferenc (2004). The (*Z*)-isomer was obtained by recrystallization of the isomeric mixture from hexanes/ethyl acetate. Single crystals suitable for X-ray crystallography were grown by evaporation from a CH_2_Cl_2_ solution.

### Refinement

Hydrogen atoms attached to carbons were placed at calculated positions with C—H = 0.95 Å (aromatic) or 0.98–0.99 Å (*sp*^3^ C-atom). Hydrogen atoms attached to N were located in difference Fourier maps. All H atoms were refined in the riding-model approximation with isotropic displacement parameters (set at 1.2–1.5 times of the *U*_eq_ of the parent atom). The crystal used for data collection was twinned. Using the twin matrix 0 - 1 0, -1 0 0, 0 0 - 1 with BSAF 0.468 (1) the structure refinement was succesful.

### Crystal data

**Table:**

C_11_H_9_N_3_O	*Z* = 4
*M**_r_* = 199.21	*F*(000) = 416
Triclinic, *P*1	*D*_x_ = 1.429 Mg m^−^^3^
Hall symbol: -P 1	Mo *K*α radiation, λ = 0.71073 Å
*a* = 8.5021 (7) Å	Cell parameters from 5592 reflections
*b* = 8.5080 (6) Å	θ = 2.5–27.8°
*c* = 14.0412 (11) Å	µ = 0.10 mm^−^^1^
α = 80.741 (5)°	*T* = 298 K
β = 80.797 (5)°	Block, yellow
γ = 68.329 (4)°	0.44 × 0.30 × 0.24 mm
*V* = 925.98 (12) Å^3^	

### Data collection

**Table:**

Bruker SMART APEXII diffractometer	3668 reflections with *I* > 2σ(*I*)
Radiation source: sealed tube	*R*_int_ = 0.095
graphite	θ_max_ = 28.4°, θ_min_ = 2.6°
CCD scan	*h* = −11→11
13710 measured reflections	*k* = −11→11
4570 independent reflections	*l* = −18→18

### Refinement

**Table:**

Refinement on *F*^2^	Primary atom site location: structure-invariant direct methods
Least-squares matrix: full	Secondary atom site location: difference Fourier map
*R*[*F*^2^ > 2σ(*F*^2^)] = 0.047	Hydrogen site location: inferred from neighbouring sites
*wR*(*F*^2^) = 0.114	H-atom parameters constrained
*S* = 0.97	*w* = 1/[σ^2^(*F*_o_^2^) + (0.0637*P*)^2^] where *P* = (*F*_o_^2^ + 2*F*_c_^2^)/3
4570 reflections	(Δ/σ)_max_ = 0.001
274 parameters	Δρ_max_ = 0.24 e Å^−^^3^
0 restraints	Δρ_min_ = −0.29 e Å^−^^3^

### Special details

**Table:**

Geometry. All e.s.d.'s (except the e.s.d. in the dihedral angle between two l.s. planes) are estimated using the full covariance matrix. The cell e.s.d.'s are taken into account individually in the estimation of e.s.d.'s in distances, angles and torsion angles; correlations between e.s.d.'s in cell parameters are only used when they are defined by crystal symmetry. An approximate (isotropic) treatment of cell e.s.d.'s is used for estimating e.s.d.'s involving l.s. planes.
Refinement. Refinement of *F*^2^ against ALL reflections. The weighted *R*-factor *wR* and goodness of fit *S* are based on *F*^2^, conventional *R*-factors *R* are based on *F*, with *F* set to zero for negative *F*^2^. The threshold expression of *F*^2^ > σ(*F*^2^) is used only for calculating *R*-factors(gt) *etc*. and is not relevant to the choice of reflections for refinement. *R*-factors based on *F*^2^ are statistically about twice as large as those based on *F*, and *R*- factors based on ALL data will be even larger.

### Fractional atomic coordinates and isotropic or equivalent isotropic displacement parameters (Å^2^)

**Table:**

	*x*	*y*	*z*	*U*_iso_*/*U*_eq_	
C1A	0.2396 (3)	0.2747 (2)	0.03598 (13)	0.0253 (4)	
C2A	0.0717 (3)	0.2796 (3)	0.05168 (15)	0.0336 (5)	
H2A	0.0273	0.2403	0.0079	0.040*	
C3A	−0.0270 (3)	0.3451 (3)	0.13471 (15)	0.0383 (5)	
H3A	−0.1390	0.3487	0.1464	0.046*	
C4A	0.0353 (3)	0.4049 (3)	0.20035 (15)	0.0368 (5)	
H4A	−0.0345	0.4474	0.2554	0.044*	
C5A	0.2019 (3)	0.4024 (2)	0.18496 (14)	0.0331 (4)	
H5A	0.2454	0.4423	0.2289	0.040*	
C6A	0.3004 (3)	0.3385 (2)	0.10205 (13)	0.0269 (4)	
C7A	0.4779 (3)	0.3235 (2)	0.06796 (13)	0.0270 (4)	
N8A	0.5196 (2)	0.2477 (2)	−0.01665 (11)	0.0260 (3)	
C9A	0.3809 (2)	0.2157 (2)	−0.04199 (13)	0.0244 (4)	
O10A	0.5747 (2)	0.36850 (19)	0.10405 (10)	0.0367 (3)	
C11A	0.6877 (3)	0.2169 (3)	−0.07052 (14)	0.0334 (5)	
H11A	0.7539	0.2569	−0.0375	0.050*	
H11B	0.6761	0.2765	−0.1346	0.050*	
H11C	0.7439	0.0971	−0.0749	0.050*	
C12A	0.3794 (3)	0.1446 (2)	−0.12086 (13)	0.0274 (4)	
N13A	0.5046 (2)	0.1033 (2)	−0.19937 (12)	0.0378 (4)	
H13A	0.4929	0.0314	−0.2428	0.057*	
H13B	0.6248	0.0872	−0.1858	0.057*	
C14A	0.2264 (3)	0.1156 (3)	−0.13240 (14)	0.0333 (5)	
N15A	0.1094 (3)	0.0862 (3)	−0.14296 (14)	0.0497 (5)	
C1B	0.2361 (2)	0.7692 (2)	0.48593 (13)	0.0255 (4)	
C2B	0.2327 (3)	0.9368 (2)	0.46907 (14)	0.0315 (5)	
H2B	0.2776	0.9793	0.5111	0.038*	
C3B	0.1607 (3)	1.0388 (3)	0.38784 (15)	0.0365 (5)	
H3B	0.1575	1.1506	0.3760	0.044*	
C4B	0.0932 (3)	0.9777 (3)	0.32407 (15)	0.0364 (5)	
H4B	0.0454	1.0487	0.2706	0.044*	
C5B	0.0971 (3)	0.8124 (3)	0.33976 (14)	0.0340 (5)	
H5B	0.0527	0.7702	0.2975	0.041*	
C6B	0.1689 (3)	0.7110 (2)	0.42024 (13)	0.0273 (4)	
C7B	0.1883 (3)	0.5323 (2)	0.45288 (13)	0.0285 (4)	
N8B	0.2650 (2)	0.49057 (19)	0.53725 (11)	0.0283 (4)	
C9B	0.2977 (2)	0.6286 (2)	0.56289 (13)	0.0257 (4)	
O10B	0.1472 (2)	0.43614 (19)	0.41427 (10)	0.0382 (4)	
C11B	0.2984 (3)	0.3212 (2)	0.58994 (15)	0.0356 (5)	
H11D	0.2602	0.2548	0.5561	0.053*	
H11E	0.2386	0.3306	0.6540	0.053*	
H11F	0.4184	0.2667	0.5944	0.053*	
C12B	0.3717 (3)	0.6276 (2)	0.64150 (14)	0.0285 (4)	
N13B	0.4376 (3)	0.4959 (2)	0.71336 (13)	0.0409 (4)	
H13C	0.4549	0.5204	0.7730	0.061*	
H13D	0.4177	0.3943	0.7053	0.061*	
C14B	0.3965 (3)	0.7805 (3)	0.65574 (14)	0.0334 (4)	
N15B	0.4204 (3)	0.8972 (3)	0.67200 (14)	0.0483 (5)	

### Atomic displacement parameters (Å^2^)

**Table:**

	*U*^11^	*U*^22^	*U*^33^	*U*^12^	*U*^13^	*U*^23^
C1A	0.0268 (10)	0.0205 (9)	0.0297 (9)	−0.0101 (8)	−0.0021 (7)	−0.0029 (7)
C2A	0.0313 (12)	0.0356 (11)	0.0376 (10)	−0.0165 (10)	−0.0019 (9)	−0.0049 (9)
C3A	0.0289 (12)	0.0403 (12)	0.0448 (12)	−0.0153 (9)	0.0048 (9)	−0.0037 (9)
C4A	0.0401 (12)	0.0329 (11)	0.0346 (10)	−0.0133 (9)	0.0080 (9)	−0.0067 (9)
C5A	0.0417 (13)	0.0291 (10)	0.0306 (10)	−0.0159 (9)	0.0012 (9)	−0.0059 (8)
C6A	0.0340 (11)	0.0199 (9)	0.0292 (9)	−0.0135 (8)	−0.0020 (8)	−0.0011 (7)
C7A	0.0326 (10)	0.0240 (9)	0.0273 (9)	−0.0128 (8)	−0.0046 (8)	−0.0026 (7)
N8A	0.0269 (9)	0.0262 (8)	0.0288 (7)	−0.0139 (7)	−0.0008 (6)	−0.0047 (6)
C9A	0.0278 (10)	0.0203 (9)	0.0276 (9)	−0.0120 (8)	−0.0032 (7)	−0.0006 (7)
O10A	0.0405 (9)	0.0431 (8)	0.0372 (7)	−0.0240 (7)	−0.0049 (6)	−0.0111 (6)
C11A	0.0295 (11)	0.0376 (11)	0.0373 (11)	−0.0169 (9)	0.0020 (8)	−0.0095 (9)
C12A	0.0324 (11)	0.0239 (9)	0.0281 (9)	−0.0127 (8)	−0.0047 (8)	−0.0011 (7)
N13A	0.0425 (11)	0.0443 (11)	0.0347 (9)	−0.0227 (9)	0.0054 (8)	−0.0183 (8)
C14A	0.0397 (12)	0.0357 (11)	0.0288 (9)	−0.0171 (10)	−0.0050 (8)	−0.0054 (8)
N15A	0.0510 (13)	0.0666 (14)	0.0468 (11)	−0.0343 (11)	−0.0064 (9)	−0.0153 (10)
C1B	0.0258 (10)	0.0236 (9)	0.0286 (9)	−0.0122 (8)	0.0058 (7)	−0.0078 (7)
C2B	0.0379 (12)	0.0260 (10)	0.0358 (10)	−0.0179 (9)	0.0021 (9)	−0.0082 (8)
C3B	0.0420 (12)	0.0244 (10)	0.0425 (11)	−0.0148 (9)	0.0040 (9)	−0.0034 (8)
C4B	0.0419 (13)	0.0308 (11)	0.0345 (10)	−0.0130 (10)	−0.0013 (9)	0.0001 (8)
C5B	0.0379 (12)	0.0370 (11)	0.0313 (10)	−0.0183 (10)	0.0010 (8)	−0.0079 (8)
C6B	0.0316 (11)	0.0269 (9)	0.0265 (9)	−0.0153 (8)	0.0052 (7)	−0.0083 (7)
C7B	0.0337 (11)	0.0263 (10)	0.0296 (9)	−0.0159 (8)	0.0056 (8)	−0.0111 (7)
N8B	0.0372 (10)	0.0213 (8)	0.0313 (8)	−0.0159 (7)	0.0017 (7)	−0.0081 (6)
C9B	0.0273 (10)	0.0208 (9)	0.0317 (9)	−0.0118 (8)	0.0038 (7)	−0.0098 (7)
O10B	0.0548 (10)	0.0354 (8)	0.0357 (7)	−0.0260 (8)	−0.0021 (6)	−0.0137 (6)
C11B	0.0492 (13)	0.0227 (10)	0.0393 (10)	−0.0184 (9)	−0.0031 (9)	−0.0038 (8)
C12B	0.0296 (10)	0.0250 (10)	0.0332 (10)	−0.0123 (8)	0.0029 (8)	−0.0095 (7)
N13B	0.0592 (13)	0.0316 (10)	0.0369 (9)	−0.0181 (9)	−0.0131 (9)	−0.0052 (8)
C14B	0.0392 (12)	0.0346 (11)	0.0320 (10)	−0.0171 (9)	−0.0033 (8)	−0.0100 (8)
N15B	0.0668 (14)	0.0387 (11)	0.0526 (11)	−0.0287 (10)	−0.0115 (10)	−0.0127 (9)

### Geometric parameters (Å, °)

**Table:**

C1A—C2A	1.395 (3)	C1B—C6B	1.395 (3)
C1A—C6A	1.395 (3)	C1B—C2B	1.398 (3)
C1A—C9A	1.485 (2)	C1B—C9B	1.475 (3)
C2A—C3A	1.387 (3)	C2B—C3B	1.393 (3)
C2A—H2A	0.9300	C2B—H2B	0.9300
C3A—C4A	1.377 (3)	C3B—C4B	1.391 (3)
C3A—H3A	0.9300	C3B—H3B	0.9300
C4A—C5A	1.391 (3)	C4B—C5B	1.377 (3)
C4A—H4A	0.9300	C4B—H4B	0.9300
C5A—C6A	1.381 (3)	C5B—C6B	1.383 (3)
C5A—H5A	0.9300	C5B—H5B	0.9300
C6A—C7A	1.473 (3)	C6B—C7B	1.471 (3)
C7A—O10A	1.228 (2)	C7B—O10B	1.225 (2)
C7A—N8A	1.377 (2)	C7B—N8B	1.377 (3)
N8A—C9A	1.412 (2)	N8B—C9B	1.412 (2)
N8A—C11A	1.458 (2)	N8B—C11B	1.458 (2)
C9A—C12A	1.348 (3)	C9B—C12B	1.352 (3)
C11A—H11A	0.9600	C11B—H11D	0.9600
C11A—H11B	0.9600	C11B—H11E	0.9600
C11A—H11C	0.9600	C11B—H11F	0.9600
C12A—N13A	1.395 (2)	C12B—N13B	1.393 (3)
C12A—C14A	1.447 (3)	C12B—C14B	1.441 (2)
N13A—H13A	0.9688	N13B—H13C	0.9399
N13A—H13B	1.0251	N13B—H13D	0.9669
C14A—N15A	1.148 (3)	C14B—N15B	1.147 (3)
			
C2A—C1A—C6A	119.47 (17)	C6B—C1B—C2B	118.63 (19)
C2A—C1A—C9A	133.57 (17)	C6B—C1B—C9B	107.85 (15)
C6A—C1A—C9A	106.95 (16)	C2B—C1B—C9B	133.53 (18)
C3A—C2A—C1A	117.81 (19)	C3B—C2B—C1B	118.40 (18)
C3A—C2A—H2A	121.1	C3B—C2B—H2B	120.8
C1A—C2A—H2A	121.1	C1B—C2B—H2B	120.8
C4A—C3A—C2A	122.2 (2)	C4B—C3B—C2B	121.68 (18)
C4A—C3A—H3A	118.9	C4B—C3B—H3B	119.2
C2A—C3A—H3A	118.9	C2B—C3B—H3B	119.2
C3A—C4A—C5A	120.48 (19)	C5B—C4B—C3B	120.3 (2)
C3A—C4A—H4A	119.8	C5B—C4B—H4B	119.9
C5A—C4A—H4A	119.8	C3B—C4B—H4B	119.9
C6A—C5A—C4A	117.56 (19)	C4B—C5B—C6B	118.06 (19)
C6A—C5A—H5A	121.2	C4B—C5B—H5B	121.0
C4A—C5A—H5A	121.2	C6B—C5B—H5B	121.0
C5A—C6A—C1A	122.42 (18)	C5B—C6B—C1B	122.93 (17)
C5A—C6A—C7A	128.57 (17)	C5B—C6B—C7B	128.61 (17)
C1A—C6A—C7A	109.01 (16)	C1B—C6B—C7B	108.46 (17)
O10A—C7A—N8A	124.46 (18)	O10B—C7B—N8B	125.50 (18)
O10A—C7A—C6A	129.24 (18)	O10B—C7B—C6B	128.30 (19)
N8A—C7A—C6A	106.30 (15)	N8B—C7B—C6B	106.20 (14)
C7A—N8A—C9A	111.70 (15)	C7B—N8B—C9B	112.07 (15)
C7A—N8A—C11A	120.31 (15)	C7B—N8B—C11B	119.95 (14)
C9A—N8A—C11A	127.93 (15)	C9B—N8B—C11B	127.95 (16)
C12A—C9A—N8A	126.48 (17)	C12B—C9B—N8B	126.16 (17)
C12A—C9A—C1A	127.51 (17)	C12B—C9B—C1B	128.42 (16)
N8A—C9A—C1A	106.01 (14)	N8B—C9B—C1B	105.42 (15)
N8A—C11A—H11A	109.5	N8B—C11B—H11D	109.5
N8A—C11A—H11B	109.5	N8B—C11B—H11E	109.5
H11A—C11A—H11B	109.5	H11D—C11B—H11E	109.5
N8A—C11A—H11C	109.5	N8B—C11B—H11F	109.5
H11A—C11A—H11C	109.5	H11D—C11B—H11F	109.5
H11B—C11A—H11C	109.5	H11E—C11B—H11F	109.5
C9A—C12A—N13A	128.35 (17)	C9B—C12B—N13B	130.12 (17)
C9A—C12A—C14A	118.38 (17)	C9B—C12B—C14B	118.57 (18)
N13A—C12A—C14A	113.12 (16)	N13B—C12B—C14B	111.26 (17)
C12A—N13A—H13A	116.8	C12B—N13B—H13C	120.2
C12A—N13A—H13B	115.0	C12B—N13B—H13D	111.4
H13A—N13A—H13B	118.1	H13C—N13B—H13D	123.8
N15A—C14A—C12A	177.1 (2)	N15B—C14B—C12B	175.9 (2)
			
C6A—C1A—C2A—C3A	−1.5 (3)	C6B—C1B—C2B—C3B	0.7 (3)
C9A—C1A—C2A—C3A	−179.98 (19)	C9B—C1B—C2B—C3B	−178.89 (19)
C1A—C2A—C3A—C4A	0.4 (3)	C1B—C2B—C3B—C4B	−0.1 (3)
C2A—C3A—C4A—C5A	0.3 (3)	C2B—C3B—C4B—C5B	−0.3 (3)
C3A—C4A—C5A—C6A	0.1 (3)	C3B—C4B—C5B—C6B	0.2 (3)
C4A—C5A—C6A—C1A	−1.3 (3)	C4B—C5B—C6B—C1B	0.4 (3)
C4A—C5A—C6A—C7A	179.10 (18)	C4B—C5B—C6B—C7B	179.96 (19)
C2A—C1A—C6A—C5A	2.1 (3)	C2B—C1B—C6B—C5B	−0.9 (3)
C9A—C1A—C6A—C5A	−179.10 (16)	C9B—C1B—C6B—C5B	178.83 (17)
C2A—C1A—C6A—C7A	−178.31 (16)	C2B—C1B—C6B—C7B	179.50 (16)
C9A—C1A—C6A—C7A	0.5 (2)	C9B—C1B—C6B—C7B	−0.8 (2)
C5A—C6A—C7A—O10A	−2.3 (3)	C5B—C6B—C7B—O10B	1.3 (3)
C1A—C6A—C7A—O10A	178.13 (19)	C1B—C6B—C7B—O10B	−179.04 (19)
C5A—C6A—C7A—N8A	178.35 (18)	C5B—C6B—C7B—N8B	−179.13 (19)
C1A—C6A—C7A—N8A	−1.3 (2)	C1B—C6B—C7B—N8B	0.5 (2)
O10A—C7A—N8A—C9A	−177.91 (18)	O10B—C7B—N8B—C9B	179.60 (18)
C6A—C7A—N8A—C9A	1.5 (2)	C6B—C7B—N8B—C9B	0.1 (2)
O10A—C7A—N8A—C11A	−0.6 (3)	O10B—C7B—N8B—C11B	−2.1 (3)
C6A—C7A—N8A—C11A	178.87 (16)	C6B—C7B—N8B—C11B	178.37 (16)
C7A—N8A—C9A—C12A	179.28 (17)	C7B—N8B—C9B—C12B	−179.94 (18)
C11A—N8A—C9A—C12A	2.2 (3)	C11B—N8B—C9B—C12B	1.9 (3)
C7A—N8A—C9A—C1A	−1.2 (2)	C7B—N8B—C9B—C1B	−0.5 (2)
C11A—N8A—C9A—C1A	−178.31 (17)	C11B—N8B—C9B—C1B	−178.69 (17)
C2A—C1A—C9A—C12A	−1.5 (3)	C6B—C1B—C9B—C12B	−179.79 (18)
C6A—C1A—C9A—C12A	179.87 (18)	C2B—C1B—C9B—C12B	−0.2 (4)
C2A—C1A—C9A—N8A	179.0 (2)	C6B—C1B—C9B—N8B	0.8 (2)
C6A—C1A—C9A—N8A	0.4 (2)	C2B—C1B—C9B—N8B	−179.6 (2)
N8A—C9A—C12A—N13A	−7.3 (3)	N8B—C9B—C12B—N13B	2.2 (3)
C1A—C9A—C12A—N13A	173.28 (18)	C1B—C9B—C12B—N13B	−177.04 (19)
N8A—C9A—C12A—C14A	177.50 (17)	N8B—C9B—C12B—C14B	179.39 (17)
C1A—C9A—C12A—C14A	−1.9 (3)	C1B—C9B—C12B—C14B	0.1 (3)

### Hydrogen-bond geometry (Å, °)

**Table:**

*D*—H···*A*	*D*—H	H···*A*	*D*···*A*	*D*—H···*A*
N13A—H13A···N15B^i^	0.97	2.08	3.035 (3)	169
N13B—H13C···O10A^ii^	0.94	2.04	2.948 (2)	163
N13B—H13D···N13A^iii^	0.97	2.52	3.238 (3)	131

Symmetry codes: (i) *x*, *y*−1, *z*−1; (ii) −*x*+1, −*y*+1, −*z*+1; (iii) *x*, *y*, *z*+1.
